# Synthesis of Polymers with Narrow Molecular Mass Distribution through Interface-Initiated Room-Temperature Polymerization in Emulsion Gels

**DOI:** 10.3390/polym15204081

**Published:** 2023-10-13

**Authors:** Miles Pamueles Duan, Zhirong Zhou, Tan Zhang

**Affiliations:** Division of Natural and Applied Sciences, Duke Kunshan University, Kunshan 215316, Chinazhirong.zhou@dukekunshan.edu.cn (Z.Z.)

**Keywords:** narrow molecular mass distribution, radical polymerization, emulsions, room-temperature polymerization

## Abstract

Homopolymers of n-butyl acrylate, methyl methacrylate, styrene, and their random copolymers were prepared via interface-initiated polymerization of emulsion gels at 20 °C. The polymerization was conducted in a free radical polymerization manner without inert gas protection. Compared with the polymers synthesized at 60 °C, the polymerization of emulsion gels at 20 °C produced homo- and copolymers with a higher molecular mass and a narrower molecular mass distribution. The polydispersity indices for the polymers synthesized at 20 °C were found to be between 1.12 and 1.37. The glass transition temperatures for the as-synthesized butyl acrylate copolymers agree well with the prediction from the Gordon–Taylor equation. Interface-initiated room-temperature polymerization is a robust, energy-saving polymerization technique for synthesizing polymers with a narrow molecular mass distribution.

## 1. Introduction

Polymerization in emulsion systems is an important technique used to produce polymers, latexes, composites, and porous materials [1,2,3,4]. At least 20 million tons of polymer materials are produced via emulsion techniques worldwide each year [2]. Usually, emulsion polymerization is conducted at elevated temperatures (60 °C or above) in order for sufficient numbers of radicals to form through thermal decomposition of initiators. However, high-temperature polymerization destabilizes colloidal structures and consumes tremendous amounts of energy [1,5,6]. With the Paris Agreement, conducting polymerization at room temperature became a key criterion for green polymer industries [6,7]. Additionally, conducting polymerization at high temperatures is not suitable for a system consisting of temperature-sensitive molecules, such as proteins [8,9].

Room-temperature polymerization can be achieved through several initiation methods. For example, 2,2′-azobis(4-methoxy-2,4-dimethylvaleronitrile), a low-temperature initiator, decomposes at 30 °C with a half-life of 10 h [10]. Low-temperature initiators require strict regulations and safety measurements as they may explode accidentally under ambient conditions. Redox initiators can initiate polymerization at room temperature efficiently, but metal complexes are usually required [11,12,13,14]. Photo and γ-radiation are also used for initiating polymerization at room temperature [9,15,16], but they are limited by production scale and may not work for some systems [17]. The interfaces in emulsions were found to lower the activation energy for initiator decomposition, consequently initiating polymerization at room temperature [1,18]. Interface initiation does not require hazardous compounds or additional devices to conduct room-temperature polymerization in an emulsion system. Therefore, interface initiation is considered an efficient eco-friendly technique for room-temperature polymerizations.

When producing a high-performance polymer, a narrow molecular mass distribution is desired [19]. Currently, narrow molecular mass distribution is mainly achieved through controlled free radical polymerizations or living radical polymerizations [20,21,22]. Compared with free radical polymerization, the experimental requirement and costs for conducting living radical polymerization are remarkably high [21]. A robust, energy-efficient, and cost-effective technique for producing polymers with a narrow molecular mass distribution is important for green polymer industries. 

**Figure 1:**
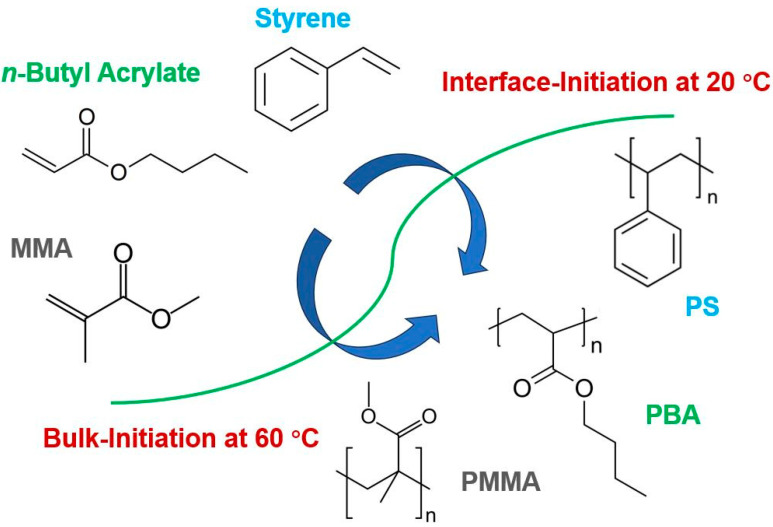
The structures of styrene, n-butyl acrylate (BA), methyl methacrylate (MMA), and their corresponding homopolymers.

Herein, we report using emulsion gels as polymerization media to produce vinyl homopolymers and copolymers with a narrow molecular mass distribution at room temperature (20 °C). The room-temperature polymerization was achieved through the thermal decomposition of 2,2′-azobisisobutyronitrile (AIBN) at emulsion interfaces [1]. For comparison, the polymers synthesized in emulsion gels at 60 °C were also characterized as demonstrated in Figure 1. This work not only demonstrates the effectiveness of conducting room-temperature polymerization in emulsion systems but also provides a cost-effective technique for producing polymers with a narrow molecular mass distribution.

## 2. Results and Discussion

**Figure 2:**
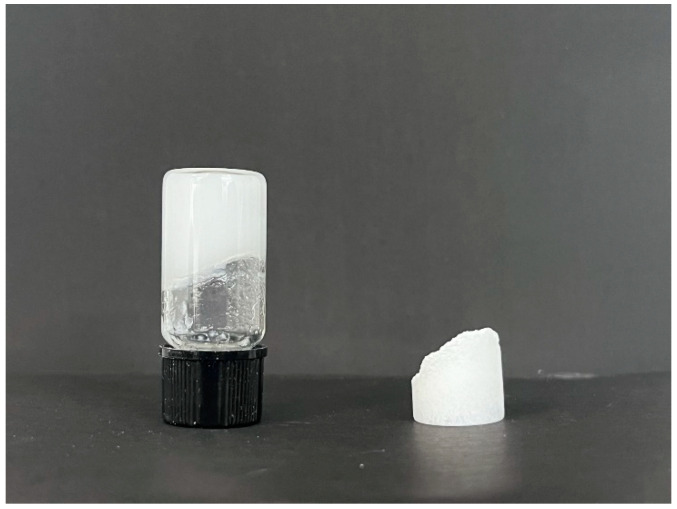
Appearance of the emulsion gel and the as-synthesized polymer composite monolith.

Increasing emulsion viscosity stabilizes oil–water interfaces, which enhances interface initiation at room temperature [1,23]. Emulsion gels were obtained by adding fumed silica particles into emulsions. Chain-like fumed silica particles bridged monomer-dispersed phases in emulsions to form a three-dimensional network, leading to gelation in emulsions or emulsion gels [5,24]. All the emulsions used in this study were in their gel state, as seen in Figure 2. Different monomers did not affect the gel stability with the compositions used in this study. The emulsion gels remained stable without phase separation for weeks in ambient conditions. 

The polymerizations of emulsion gels at 20 °C and 60 °C were conducted as free radical polymerization without inert gas protection. Initiator AIBN decomposed efficiently at emulsion interfaces at 20 °C, consequently initiating polymerization [18,23]. At 60 °C, thermal initiation of AIBN in the bulk phase occurred [1]. A monomer-to-polymer conversion of 70% was reached within 6 h for most of the polymerizations conducted at 60 °C and 5–7 days for those conducted at 20 °C. For *n*-butyl acrylate (BA), the conversion at 60 °C only reached 50% within 6 h due to the relatively low reactivity of BA [25,26]. Slower polymerizations at 20 °C are likely because only the AIBN molecules at the interfaces are decomposed [1,18,27]. Although AIBN keeps migrating to the interfaces [18], the overall initiation rate at 20 °C is smaller compared with that at 60 °C [23]. A reduced initiation efficiency (<0.4) at 20 °C also contributes to the smaller polymerization rate [28]. The resulting monoliths are white and uniform in their appearance (Figure 2). The nuclear magnetic resonance spectra for the extracted polymers and their tacticity analysis can be found in the Supplementary Materials.

The differences in molecular mass were observed for the extracted polymers synthesized at different temperatures. As seen in Table 1, the weight average molecular masses for poly(butyl acrylate) (PBA), polystyrene (PS), poly(methyl methacrylate) (PMMA), and butyl acrylate copolymers synthesized at 20 °C are significantly greater than those of their counterparts synthesized at 60 °C. For example, the PBA synthesized at 60 °C is glue-like due to its very small molecular mass of 31 kg/mol. The PBA synthesized at 20 °C has a much greater molecular mass (757 kg/mol) and appears to be a rubbery material with a well-defined shape. The molecular masses and the PDI values for the homo- and copolymers synthesized at 60 °C are in line with those synthesized using conventional free radical polymerizations [29]. This suggests that emulsion gel itself has little effect on polymerizations conducted at high temperatures. 

Polymerization in an emulsion system is primarily carried out in micelles. As the combination is the probable termination mechanism [30], the number of propagating chains and their probability of collision within micelles are reduced. Compared with other polymerization techniques, polymers with a higher molecular mass can be produced using emulsion polymerization [2,31]. Based on the decomposition rate constants for the AIBN in emulsion gels (*k*_d_ ~ 10^−8^ and 10^−6^ s^−1^ at 20 °C and 60 °C, respectively) [23,32,33,34], the concentration of radicals generated at 20 °C is much smaller than that of radicals produced at 60 °C. With a reduced radical concentration, the number of propagating chains is considerably smaller in emulsion gels at 20 °C. Additionally, a highly viscous environment in emulsion gels also decreases polymer chain mobilities as the termination is diffusion-controlled [35,36]. The propagating chains in emulsion gels have a much smaller probability of colliding with each other or terminating at 20 °C. Polymer chains grow to a considerable length until the polymer particles become too large to stabilize [37]. As a result, polymers with a high molecular mass were produced in emulsion gels at 20 °C.

**Figure 3:**
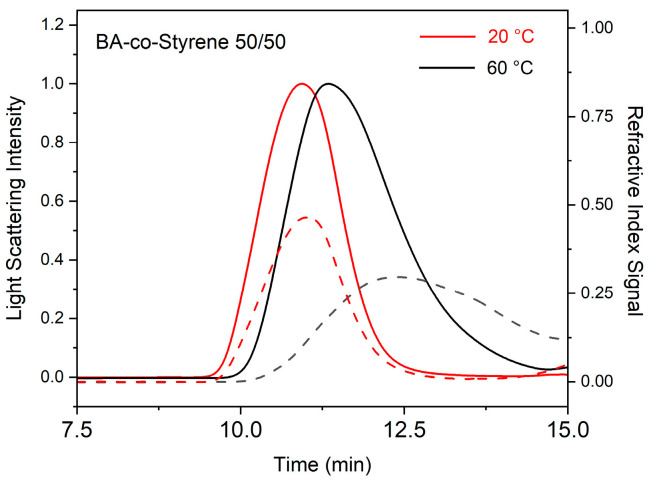
The light-scattering detector intensity (solid line) and the refractive index detector intensity (dashed line) obtained from gel permeation chromatography as a function of the elution time for the extracted butyl acrylate-co-styrene 50/50 polymers synthesized at 20 °C and 60 °C.

The polydispersity indices (PDIs) for the polymers synthesized at 20 °C were found to be between 1.12 and 1.37, which are in line with those synthesized from living radical polymerizations [38,39]. The GPC traces of the polymers synthesized in emulsion gels are shown in Figure 3 and the Supporting Information. The polymerization in emulsion gels did not proceed in a living radical manner. No known living radical agent was included in the emulsion gels. Only a trace amount of calcium was detected in the fumed silica used in this study, which rules out the possibility that the transition metal impurities alter the kinetics of the polymerization in emulsion gels. The results from inductively coupled plasma atomic emission spectroscopy (ICP) can be found in the Supporting Information. One possible reason for the obtained narrow molecular mass distribution may be the less pronounced side reactions at 20 °C. For example, *β*-scission and intramolecular transfer can alter the structure and the chain length of the resulting polymers at high temperatures [30,40]. These side effects are greatly reduced at 20 °C because of their high activation energies [30].

Low polymerization temperature should not be considered as the only reason contributing to narrow distribution. Room-temperature interface-initiated polymerization in emulsions without fumed silica produced the PS with a PDI value around 3~4 [5]. Similar results were also reported for room-temperature emulsion polymerization initiated by photo- or redox initiators, where PDI values of 2~12 were observed for PS and butyl acrylate copolymers [31,41,42]. Similar to the reason leading to high molecular masses, the relatively small radical concentrations from the interface initiation and the high viscosity in the emulsion gels greatly reduce the probability of chain termination.

The stability of silica gels increases as the temperature decreases [43]. The propagating chains can grow without early termination until the polymer particles reach their critical size for super swelling [37]. Once the critical size has been reached, the polymer particles are no longer stable in emulsion gels and tend to precipitate. As the number of the propagating chains is considerably small in the polymerization of emulsion gels at 20 °C, most of the polymer chains reach their maximum lengths and consequently produce a narrower molecular mass distribution. In sum, the narrow molecular mass distribution of the polymers synthesized in emulsion gels at 20 °C is the result of synergistic effects from a low temperature, small radical concentration, and high viscosity.

The role of fumed silica particles in the polymerization of emulsion gels is somewhat complicated. On the one hand, fumed silica induces gelation and stabilizes the oil–water interfaces in emulsion gels [5,44,45]. It facilitates AIBN decomposition and reduces the probability of propagating chain collision. On the other hand, the silanol groups on fumed silica surfaces react with radicals [32]. In the presence of silica, the PDI values generally increase for polymers synthesized using bulk polymerization, especially those synthesized via living radical polymerization [46,47,48]. In contrast, the cationic surfactants, e.g., cetyltrimethylammonium bromide (CTAB), can adsorb onto silica surfaces in emulsion gels and block the negatively charged silanol groups [32]. As a result, the side reactions between silanol groups and the propagating chains, which broaden the molecular mass distribution, are minimized in emulsion gels [32]. 

**Figure 4:**
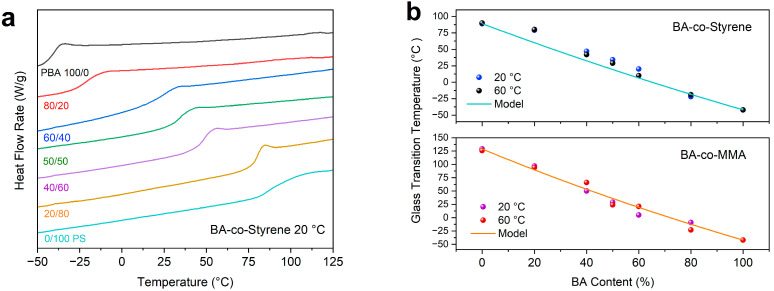
(**a**) Differential scanning calorimetry thermogram of the BA-co-styrene copolymers synthesized at 20 °C; (**b**) glass transition temperatures of the BA-co-styrene and BA-co-MMA copolymers.

The glass transition temperatures (*T*_g_) for the extracted polymers were determined from differential scanning calorimetry (DSC) thermograms. The glass transition temperatures for the polymers synthesized at 20 °C and 60 °C are close to each other (Figure 4b). This suggests that the compositions of the copolymers are less sensitive to polymerization temperature. The reactivity ratios of a monomer are dependent on temperature, i.e., the lower the temperature, the smaller the reactivity ratio [25]. For the monomers used in this study, their reactivity decreased with decreasing temperature at a similar scale [25]. Although the overall polymerization rate decreased, the ratios at which different monomers reacted with a propagating chain remained similar at 20 °C and 60 °C. The compositions of the as-synthesized copolymer and consequently the glass transition temperatures remained similar.

The thermograms for BA-co-styrene copolymer and the respective homopolymers (PBA and PS) synthesized at 20 °C are plotted in Figure 4a. PBA and PS showed a glass transition at −42 °C and 89 °C, respectively. For BA-co-styrene copolymers, the glass transition temperatures gradually increased with increased styrene content in the polymerization. The glass transition temperatures of the copolymers with different compositions are compared with the theoretical results predicted using Gordon–Taylor equation [49], or

(1) $$T_{g}=\frac{T_{g,BA}w_{BA}+KT_{g,2}w_{2}}{w_{BA}+Kw_{2}}$$

where *T*_g,*BA*_ and *T*_g,2_ are the glass transition temperatures for the homopolymer of BA and the second monomer (styrene or MMA), respectively. *w_BA_* and *w*_2_ are the weight fractions for BA and the second monomer in their copolymers, respectively. *K* is the Gordon–Taylor coefficient, which is related to the volume expansion during glass transition. The value of *K* is 0.86 for BA-co-styrene copolymers and 0.82 for BA-co-MMA copolymers [50]. The glass transition temperatures of the copolymers generally fit well with the Gordon–Taylor model, as seen in Figure 4b. This suggests that the copolymerization in emulsion gels was conducted similarly to the conventional copolymerizations.

## 3. Conclusions

We have shown the effectiveness of using interface-initiated polymerization in emulsion gels to produce homo- and copolymers at 20 °C. A low temperature, small radical concentration, and high-viscosity environment lead to the formation of polymers with a high molecular mass and narrow molecular mass distribution. The composition of the resulting copolymers is less sensitive to the polymerization temperature. Polymerization in emulsion gels provides not only an energy-efficient technique but also a robust method of producing polymers with a narrow molecular mass distribution.

## 4. Experimental Section

Styrene and methyl methacrylate were purchased from Aldrich, Darmstadt, Germany, and *n*-butyl acrylate was obtained from Greagent, Shanghai, China. All monomers were purified by passing them through a base-activated alumina column before use. 2,2′-azobisisobutyronitrile (Greagent, Shanghai, China) was purified via recrystallization from methanol and dried in a vacuum oven for 12 h to eliminate solvent residue. Cetyltrimethylammonium bromide (Aldrich, Delhi, India) and fumed silica (Cab-O-Sil M5, Cabot, Inner Mongolia, China) were used as received. 

Emulsion gels were prepared using the following procedure: 2.0 mL of monomer, 0.105 g of fumed silica, and 0.04 g of AIBN were mixed in a glass vial first, and then 0.5 mL of 0.5 M CTAB aqueous solution was added. The mixtures were mixed using a vortex mixer for 1 min to form stable emulsion gels. The polymerization of the emulsion gels was conducted in an ambient, dark environment at 20 °C or 60 °C. After the completion of the polymerization, the polymerized samples were dried under ambient conditions and then under vacuum to remove water and unreacted monomers. The conversions were determined by comparing the sample masses before and after the polymerization by subtracting the masses of fumed silica and CTAB. 

For polymer extraction, the as-synthesized monoliths were dissolved in toluene. A methanol solution (methanol/deionized water: 85/15 by volume) was used to precipitate polymers from toluene. The extracted polymers were washed with methanol and dried under vacuum before characterization.

The molecular mass and the polydispersity index were measured in tetrahydrofuran using gel permeation chromatography (Waters E2695, Milford, MA, USA) with a Heleos light-scattering detector and a refractive index detector (Optilab rEX, Phoenix, AZ, USA). Differential scanning calorimetry measurements were taken using a DSC 25 (TA Instruments, New Castle, DE, USA) with a scan rate of 10 °C/min. The thermograms from the second heating procedure were plotted. Inductively coupled plasma–atomic emission spectroscopy (ICP) was measured using a Avio 200 (PerkinElmer, Waltham, MA, USA). 

## Figures and Tables

**Table 1:** The molecular mass and polydispersity index (PDI) of the extracted polymers from emulsion gels.

Polymer	20 °C		60 °C	
	Mw (kg/mol)	PDI	Mw (kg/mol)	PDI
PBA	757	1.26	31	2.46
PS	1661	1.37	172	2.04
PMMA	674	1.12	108	3.86
Butyl acrylate-co-styrene 50/50	1807	1.17	96	3.06
Butyl acrylate-co-MMA 50/50	888	1.25	257	1.96

## Data Availability

The raw data are available upon request from the corresponding author.

## Supplementary Materials

The following supporting information can be downloaded at: https://www.mdpi.com/article/10.3390/polym15204081/s1, S1: Nuclear magnetic resonance (NMR) spectra for the polymers synthesized at 20 °C; S2: GPC traces for the polymers synthesized at 20 °C; S3: Composition of fumed silica. Reference [51] is cited in the Supplementary Materials.
