# Supplementary material for: Synthesis of Polymers with Narrow Molecular Mass Distribution through Interface-Initiated Room-Temperature Polymerization in Emulsion Gels
Source: Polymers (Basel). 2023 Oct 13;15(20):4081. doi: 10.3390/polym15204081 (PMC10610333; doi:10.3390/polym15204081)
Supplement: Supplementary file 1 [file polymers-15-04081-s001.zip › polymers-2640504-supplementary.pdf]

Supplementary Materials

Synthesis of Polymers with Narrow Molecular Mass Distribution through Interface-Initiated Room-Temperature Polymerization in Emulsion Gels

Miles Pamueles Duan †, Zhirong Zhou † and Tan Zhang *

Division of Natural and Applied Sciences, Duke Kunshan University, Kunshan 215316, China; zhirong.zhou@dukekunshan.edu.cn (Z.Z.)

* Correspondence: tan.zhang@dukekunshan.edu.cn

† These authors contributed equally to this work.

S1 Nuclear magnetic resonance (NMR) spectra for the polymers synthesized at 20 °C

The NMR spectra for the extracted homopolymers synthesized at 20°C are plotted in Figure S1. The tacticity of PMMA was determined by comparing the peaks at 0.8, 1.0, and 1.2 ppm, corresponding to <*rr*>, <*mr*>, and <*mm*> triads [24]. This suggests that the PMMA obtained at 20°C is syndiotactic-biased. Similar tacticity was also observed for PBA. It is well known that polymerization at lower temperatures favors the formation of syndiotactic acrylate polymers [51]. For PS, the tacticity was found to be atactic, which agrees with the results for the PS synthesized at room temperature [5].

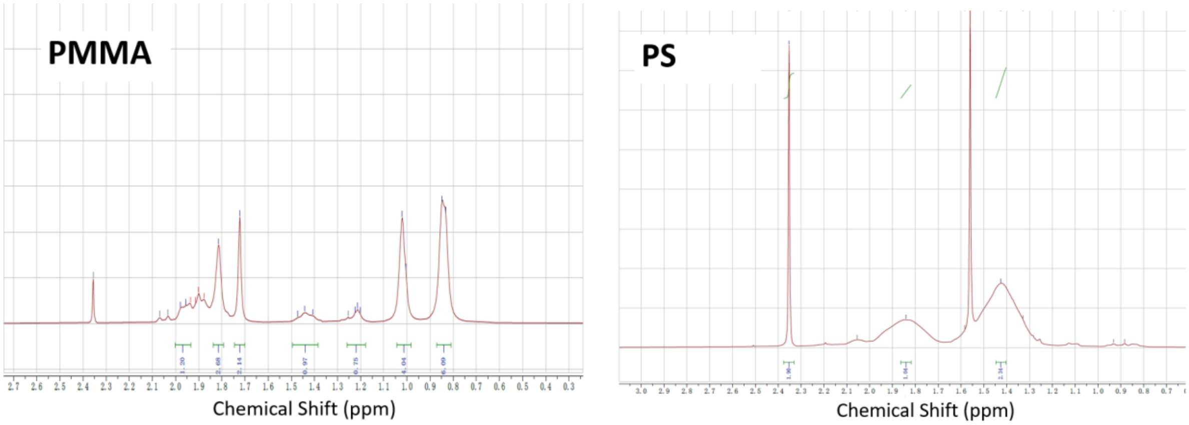

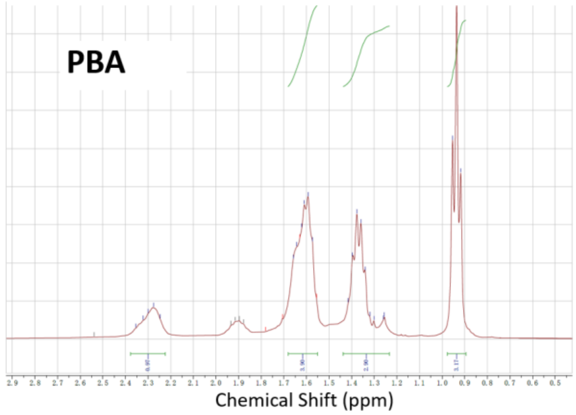

Figure S1. The NMR spectra for the extracted homopolymers synthesized at 20°C.

S2 GPC traces for the polymers synthesized at 20°C

The additional GPC traces for the polymers synthesized at 20°C are plotted in Figure S1. For poly(methyl methacrylate), polystyrene, poly(butyl acrylate), and butyl acrylate-co-methyl methacrylate copolymers, the GPC traces suggested a narrow molecular mass distribution based on the results from both light-scattering and refractive index detectors.

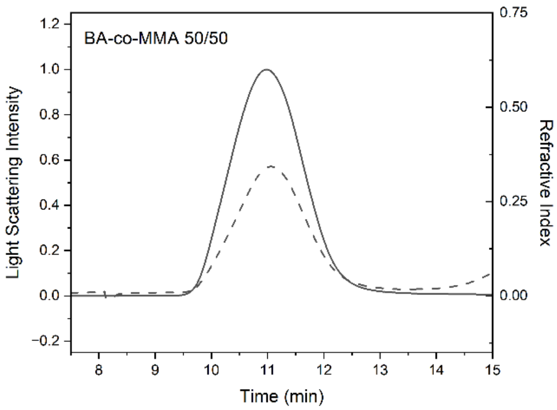

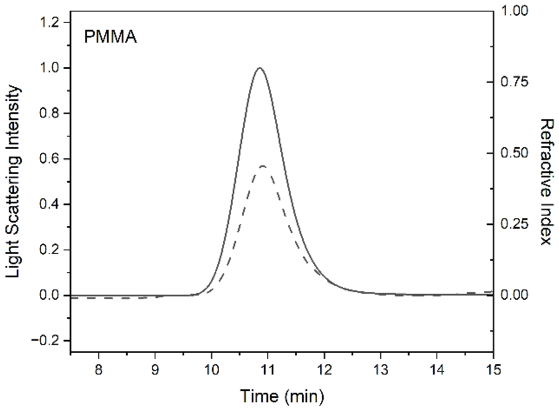

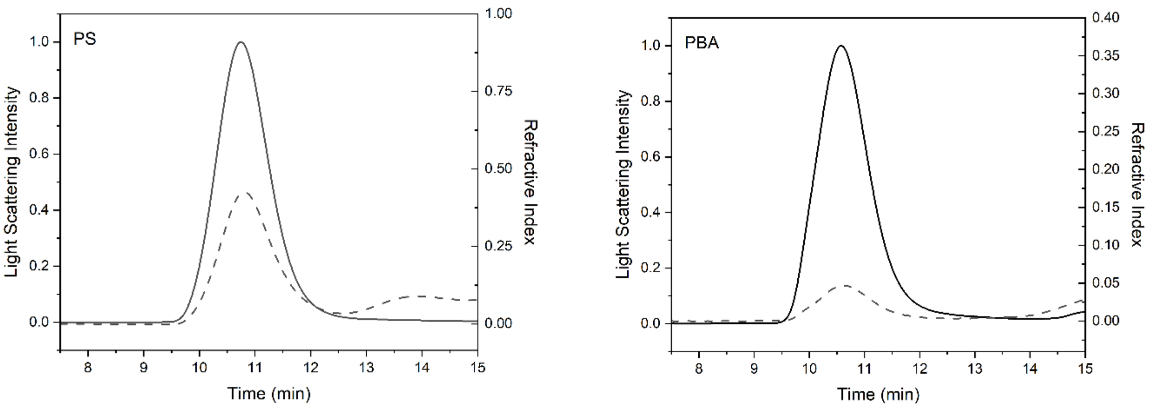

Figure S2. The light-scattering detector intensity (solid line) and the refractive index detector intensity (dash line) obtained from gel permeation chromatography as a function of the elution time for the extracted polymers synthesized at 20°C.

S3 Composition of fumed silica

The possible metallic impurities in the fumed silica used in this study were characterized using inductively coupled plasma–atomic emission spectroscopy (ICP). The detection limit for the ICP test was 10.0 mg/kg. As seen in Table S1, only a trace amount of calcium, 383.3 mg/kg, was detected. Calcium compounds usually do not act as a catalyst in radical reactions. Therefore, the observed polymerization results could not have originated from the metal impurities in the fumed silica.

Table S1. The metallic element composition in the fumed silica tested using ICP.

Element	Result	Element	Result	Element	Result
Si	464403.8	Ga	ND	Pb	ND
Ca	383.3	Ge	ND	Pd	ND
Ag	ND	Hg	ND	Pt	ND
Al	ND	Hf	ND	Ru	ND
As	ND	In	ND	Sb	ND
Au	ND	Ir	ND	Se	ND
B	ND	K	ND	Sn	ND
Ba	ND	La	ND	Sr	ND
Be	ND	Li	ND	Ta	ND
Bi	ND	Mg	ND	Te	ND
Cd	ND	Mn	ND	Ti	ND
Ce	ND	Mo	ND	Tl	ND
Co	ND	Na	ND	V	ND

Cr	ND	Nd	ND	W	ND
Cu	ND	Ni	ND	Zn	ND
Fe	ND	P	ND	Zr	ND

ND: not detected.

References

5. Zhang, T.; Xu, G.; Li, Z.F.; Regev, O.; Maddumaarachchi, M.; Blum, F.D. PS/CTAB/silica composites from room temperature polymerization of high internal phase emulsion gels. *J. Colloid Interface Sci.* **2015**, *451*, 161.

24. Zhang, T.; Xu, G.; Regev, O.; Blum, F.D. Low-temperature polymerization of methyl methacrylate emulsion gels through surfactant catalysis. *J. Colloid Interface Sci.* **2016**, *461*, 128.

51. Miyake, G.M.; Chen, E.Y.-X. Synthesis of highly syndiotactic polymers by discrete catalysts or initiators. *Polym. Chem.* **2011**, *2*, 2462.
